# Drug-Loaded Lipid-Coated Hybrid Organic-Inorganic “Stealth” Nanoparticles for Cancer Therapy

**DOI:** 10.3389/fbioe.2020.01027

**Published:** 2020-09-15

**Authors:** Xue Li, Giuseppina Salzano, Jingwen Qiu, Mathilde Menard, Kristian Berg, Theodossis Theodossiou, Catherine Ladavière, Ruxandra Gref

**Affiliations:** ^1^Université Paris-Saclay, CNRS UMR 8214, Institut des Sciences Moléculaires d’Orsay, Orsay, France; ^2^Department of Radiation Biology, Institute for Cancer Research, Oslo University Hospital, Oslo, Norway; ^3^University of Lyon, CNRS, UMR 5223, IMP, Villeurbanne, France

**Keywords:** metal organic frameworks, nanoparticles, lipids, poly(ethylene glycol), stealth, sustained drug release

## Abstract

Hybrid porous nanoscale metal organic frameworks (nanoMOFs) made of iron trimesate are attracting increasing interest as drug carriers, due to their high drug loading capacity, biodegradability, and biocompatibility. NanoMOF surface modification to prevent clearance by the innate immune system remains still challenging in reason of their high porosity and biodegradable character. Herein, FDA-approved lipids and poly(ethylene glycol) (PEG)-lipid conjugates were used to engineer the surface of nanoMOFs by a rapid and convenient solvent-exchange deposition method. The resulting lipid-coated nanoMOFs were extensively characterized. For the first time, we show that nanoMOF surface modification with lipids affords a better control over drug release and their degradation in biological media. Moreover, when loaded with the anticancer drug Gem-MP (Gemcitabine-monophosphate), iron trimesate nanoMOFs acted as “Trojan horses” carrying the drug inside cancer cells to eradicate them. Most interestingly, the PEG-coated nanoMOFs escaped the capture by macrophages. In a nutshell, versatile PEG-based lipid shells control cell interactions and open perspectives for drug targeting.

## Introduction

Despite progresses in drug development and cancer biology, cancer mortality rate remains over 30%, and the morbidity much higher. Nanomedicine has shown great promise through drug delivery by achieving drug transcytosis, drug targeting and theranostics (Rosenblum et al., 2018; Senapati et al., 2018). Nanoscale metal organic frameworks (nanoMOFs) recently emerged as an attracting class of hybrid nanomaterials for biomedical applications due to their biodegradability, biocompatibility, elevated drug loading capacity and high versatility in terms of architecture and physico-chemical properties (Horcajada et al., 2010, 2012; He et al., 2015; Rojas et al., 2019). NanoMOFs are formed by the self-assembly of metal centers and organic ligands, leading to the formation of open crystalline structures with regular and high porosities.

**FIGURE 1:**
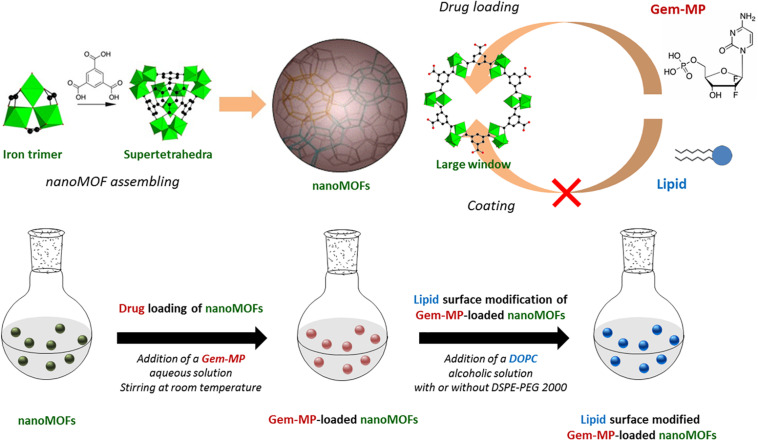
**Upper panel**: Schematic representation of the MIL-100(Fe) nanoMOF assembly from iron trimers and trimesic acid. Gem-MP was loaded by impregnation from aqueous solutions, penetrating inside the nanoMOFs through their largest windows (approx. 9 Å in size). Lipid molecules (DOPC and DSPE-PEG 2000) with larger molecular dimensions than the large windows were used to coat the nanoMOFs. **Lower panel**: Preparation steps of lipid-coated nanoMOFs. First, Gem-MP was loaded, into the nanoMOFs followed by their coating with lipid shells and PEG-lipid conjugates.

Iron (III) trimesate nanoMOFs (Figure 1 upper panel) are among the most widely studied MOFs for drug delivery (Horcajada et al., 2010; Agostoni et al., 2013; Baati et al., 2013; Simon-Yarza et al., 2016; Li et al., 2019a). Recently, they were shown to display several intrinsic properties of main interest in the nanomedicine field: radio-enhancement properties when submitted to γ-irradiation (Li et al., 2019a); they behaved as T_2_-weighted MRI imaging contrast agents (Horcajada et al., 2010) and they had intrinsic antibacterial effects killing intracellular bacteria (Li et al., 2019b).

In addition, iron trimesate nanoMOFs MIL-100 (Fe) (MIL stands for Material from Institut Lavoisier) exhibited high drug loading capacity soaking a variety of drugs from their aqueous solutions with yields close to 100%. In the case of Gemcitabine-monophosphate (Gem-MP), the drug payload reached ∼30 wt% with >98% drug loading efficiency (Rodriguez-Ruiz et al., 2015). Gem-MP, the active intermediate of Gem, is widely used in various carcinomas, including pancreatic cancer, bladder cancer, and non-small cell lung cancer. The administration of Gem-MP is of high interest for resistant cancer treatment since the phosphorylation of Gem can be a rate-limiting step especially difficult for resistant cancer cells. However, Gem-MP administration is hampered by its poor stability in biological media and low cellular uptake (Bouffard et al., 1993). In this challenging context, some of us showed that Gem-MP could be protected against degradation with increased cellular uptake by encapsulation in nanoMOFs (Rodriguez-Ruiz et al., 2015).

Surface modifications are essential to control drug release and modulate the *in vivo* fate of nanoMOFs in the living body. Silica coatings were employed in an attempt to control the release of loaded molecules from nanoMOFs MIL-101 (Taylor-Pashow et al., 2009). NanoMOFs were coated with lipid bilayers to improve their uptake by cancer cells (Wuttke et al., 2015) or with chitosan to increase their intestinal permeability (Hidalgo et al., 2017). Heparin coatings endowed the nanoMOFs with longer-blood circulation time (Bellido et al., 2015).

Poly(ethylene glycol) (PEG) based materials remain the most employed ones to engineer coatings able to prevent nanoparticles (NPs) clearance by the innate immune system, which is a prerequisite for biomedical applications (Gref et al., 1994, 1995). However, as compared to dense polymeric NPs, the porous nanoMOFs are more challenging to be coated with PEG, because these linear chains readily penetrate within their porosity, inducing an uncontrolled “burst” drug release (Agostoni et al., 2015). There are still scarce examples of successful PEGylated nanoMOF formulations. PEG was crosslinked onto the nanoMOF’s surface to avoid its penetration inside the porous cores (Giménez-marqués et al., 2018) but resulted in a non-biodegradable coating. Alternatively, nanoMOFs were coated with inclusion complexes consisting of functionalized cyclodextrins (CDs) and PEG chains coupled to adamantine (Agostoni et al., 2015; Aykac et al., 2017; Cutrone et al., 2019a). Finally, comb-like copolymers consisting of polysaccharides grafted with moieties able to coordinate to the nanoMOFs and PEG chains were synthesized and anchored onto the nanoMOFs (Cutrone et al., 2019b). However, all these coatings imply sophisticated chemistry strategies and/or several preparation steps, which might restrict their further applications. Moreover, all the PEGylated (macro) molecules used in the previous studies are not approved by Food and Drug Administration (FDA).

In this context, we propose to engineer for the first time PEGylated coatings on nanoMOFs by using only FDA-approved materials using a convenient one-step method. To date, Doxil R and Onivyde R represent the only FDA-approved PEGylated NPs (Barenholz, 2012), where DSPE-PEG 2000 (1,2-distearoyl-*sn*-glycero-3-phosphoethanolamine-N-[amino (poly-ethylene glycol)-2000] sodium salt) were used in both cases. Herein, DSPE-PEG 2000 was used in combination with DOPC (1,2-dioleoyl-*sn*-glycero-3-phosphocholine) to functionalize the surface of iron trimesate MOFs (Figure 1 lower panel). Moreover, we show that the PEG-based coating have an impact on both drug release and nanoMOFs degradation, which was not the case with the coatings used so far. Finally, the coatings were able not only to reduce macrophage uptake *in vitro* but also to kill cancer cells.

## Materials and Methods

### Materials

Iron (III) chloride hexahydrate (98%) was purchased from Alfa Aesar (Schiltigheim, France). 1,3,5-benzenetricarboxylic acid (BTC, 95%) and absolute ethanol (99%) were from Sigma-Aldrich (Saint-Quentin-Fallavier, France). These materials were used for the synthesis of nanoMOFs. Amoxicillin (Amox) from Sigma-Aldrich (Saint-Quentin-Fallavier, France) and 2′,2′-difluorodeoxycytidine monophosphate (Gem-MP) from Toronto Research Chemicals (North York, Canada) were the drugs used in this study. 1,2-dioleoyl-sn-glycero-3-phosphocholine (DOPC) and (1,2-distearoyl-*sn*-glycero-3-phosphoethanolamine-N-[amino(polyethylene glycol)-2000] sodium salt (DSPE-PEG 2000) were ordered from Avanti Polar Lipids (Alabama, United States) as coating materials. 3-(4,5-Dimethyl-2-thiazolyl)-2,5-diphenyl-2H-tetrazolium bromide (MTT, Sigma-Aldrich, Oslo, Norway) was used for toxicity evaluation of nanoMOFs. All the chemicals were used without further purification.

### Cell Culture

Murine macrophage cell line J774A.1, *CelluloNet biobank BB-0033-00072*, were grown in RPMI-1640 medium (Thermo Fisher Scientific, Villebon-sur-Yvette, France) supplemented with 10% v/v decomplemented fetal bovine serum (FBS, Thermo Fisher Scientific, Villebon-sur-Yvette, France), 1% L-Gluthamine (Sigma-Aldrich, Oslo, Norway), and 1% (P/S, Sigma-Aldrich, Oslo, Norway) at 37°C in humidified air containing 5% CO_2_. SKOV3 ovarian cancer cell were cultivated in a RPMI-1640 media without phenol red supplemented with 10% FBS, 5% L-Gluthamine and 5% penicillin/streptomycin (P/S) at 37°C in a 5% CO_2_ humidified atmosphere.

### Synthesis and Characterization of MIL-100(Fe) NanoMOFs

Iron trimesate nanoMOFs was synthesized by microwave assisted hydrothermal reaction as previously described [6]. Briefly, 20 mL of aqueous mixture containing 6.0 mM of iron chloride hexahydrate and 4.02 mM of trimesic acid (TA, 1,3,5-benzenetricarboxylic acid) was heated at 130°C for 6 min under stirring. The reaction was carried out with the power of 1600 W (Mars-5, CEM, United States). The as-synthesized nanoMOFs were harvested by centrifugation (10,000 *g*, 15 min) and washed with absolute ethanol to remove the excessive TA until the supernatant became colorless. NanoMOFs were stored in ethanol at room temperature for further usage at the concentration of 18.2 mg/mL.

SEM images were acquired on a Zeiss SUPRA 55 VP field emission gun scanning electron microscope fitted with an EDAX EDS analytical system. It was set to a low voltage (1 kV) and low current (a few pA) in order not to damage the samples and to avoid any conductive coating that could bother direct observation of the samples. Secondary electron type detector was used to record the images.

Dynamic light scattering (DLS) measurements were performed at 25°C on a Malvern Zetasizer Nano-ZS instrument at 90° angle. The mean hydrodynamic diameter of the particles was determined in a diluted aqueous suspension at 50 μg/mL.

Nanoparticle tracking analysis (NTA) was performed on Malvern NanoSight (LM10 Instrument, Malvern Instruments Ltd., Orsay, France), which combines a conventional optical microscope with a laser to illuminate the NPs in Brownian motion. It is used to individually follow nanoMOFs to gain insight into their size distribution and concentration.

Zeta potential (ZP) of nanoMOFs were measured at 25°C using a Zetasizer Nano-ZS instrument at different pH ranging from 3 to 10. NanoMOFs was diluted to 100 μg/mL with 1 mM KCl. Measured electrophoretic mobilities were converted to zeta potential values according to the Smoluchowski equation. Nitrogen sorption measurements were performed on a Micromeritics Instruments ASAP 2020 at 77 K. Samples were degassed at 100°C for 15 h. BET surface area was calculated in the partial pressure range of 0.05 – 0.20 P/P_0_.

### Drug Encapsulation in NanoMOFs

Drugs (Gem-MP and Amox) were loaded within nanoMOFs simply by impregnation of drug(s) aqueous solutions and nanoMOFs. Practically, nanoMOFs suspension (1.0 mg) were centrifuged for 10 min at 10,000 *g* and re-suspended in 1 mL of aqueous drug solutions (0.125 ∼ 1 mg/mL for Amox and 0.08 ∼ 0.2 mg/mL for Gem-MP) or water as a control. Different drug concentrations were used to optimize the drug encapsulation. After incubation at room temperature under gentle stirring for several hours (12 h for Amox and 4 h for Gem-MP), the nanoMOFs were recovered by centrifugation at 10,000 *g* for 10 min. The non-encapsulated drug in the supernatant was quantified by adapting previously described High Performance Liquid Chromatography (HPLC) methods (Li et al., 2019a, b). Specifically, HPLC analysis was performed on an Agilent system using a tunable UV absorbance detector. The injection volume of AMOX was 10 μL followed by eluant flow at a rate of 0.5 mL/min through a C18 Silica column (4.6 × 250 mm, 5 μm; Phenomenex) maintained at 30°C. The mobile phase consisted of 30% (v/v) methanol containing 5.2 mg/mL of sodium dihydrogene phosphate monohydrate. The pH was adjusted to 5 using phosphoric acid solution. AMOX were detected at 247 nm and retention times were 4.6 min. Similarly, Gem-MP was detected using the same Agilent system and column. The mobile phase was composed of 84% buffer [0.2 M (TEAA)]: 16% methanol. It was detected at 254 nm with an injection volume of 10 μl. The drug payload was calculated as Equation (1):

(1) $$Payload\left(\%\right)=\frac{E\,n\,c\,a\,p\,s\,u\,l\,a\,t\,e\,d\,D\,r\,u\,g\,\left(m\,g\right)\,}{n\,a\,n\,o\,M\,O\,F\,s\,\left(m\,g\right)}\times 100$$

### Surface Modification of NanoMOFs With DOPC Lipids and PEG-Lipid Conjugates

Surface modification was performed using a “green” method. To prepare DOPC coated nanoMOFs, 60 μl of nanoMOFs were mixed with 40 μl of DOPC alcoholic solution containing 100 μg of DOPC. Subsequently, 900 μl of water were rapidly added using an electronic pipette. The weight ratio between DOPC and nanoMOF was in the range of 1:20 ∼ 1:1. In the case of PEG-lipid conjugates coated nanoMOFs, 20 wt% of DOPC was replaced by DSPE-PEG 2000.

### Characterization of Lipid Coated NanoMOFs

#### Lipid Quantification

DOPC quantification was performed by a colorimetric, enzymatic method (BIOLABO, Maizy, France) which is commonly used to determine the phospholipid amount in serum. This titration is based on the assay of the choline moiety of phospholipids. To do this, 10 μL of specimens or a standard solution were mixed with the reagents in the BIOLABO titration kit. The mixtures were stirred 10 min. at 37°C. Then, the absorbance at 500 nm of all samples was measured. The DOPC concentration was finally calculated as Eq. (2):

(2) $$D\,O\,P\,C\,c\,o\,n\,c\,e\,n\,t\,r\,a\,t\,i\,o\,n=S\,t\,a\,n\,d\,a\,r\,d\,c\,o\,n\,c\,e\,n\,t\,r\,a\,t\,i\,o\,n\times \frac{A\,b\,s\,\left(s\,p\,e\,c\,i\,m\,e\,n\right)}{A\,b\,s\,\left(s\,t\,a\,n\,d\,a\,r\,d\right)}\times 100$$

#### NPs Concentration Measurements by NP Tracking Analysis (NTA)

The concentration of nanoMOFs modified with DOPC or PEG-lipid conjugates at different weight ratios was investigated by Nanosight (LM10 Instrument, Malvern Instruments Ltd., Orsay, France), which combines a conventional optical microscope with a laser to illuminate the NPs in Brownian motion. Of main interest here, the size distribution and concentration could be determined simultaneously. Results are expressed as means of five independent measurements.

#### Colloid Stability Characterization by DLS

The colloid stability of the nanoMOFs before and after lipid surface modification was monitored in water every day during 3 weeks’ storage at 4°C. The stability in biological medium, including cell culture medium and phosphate buffer saline (PBS) used in this study, was also measured at 0, 0.5, 1, 2, 4, 6, and 8 h after incubation at 37°C.

#### Drug Release and Degradation of nanoMOFs

Drug release was performed in PBS of different concentrations at 37°C. Briefly, drug loaded nanoMOFs were centrifuged at 10,000 *g* for 10 min and the pellet was re-dispersed in 1 mL water by vortex. Aliquots of 100 μL were taken and mixed with 900 μl of the media used for release. The final concentration of PBS was 1, 3, and 6 mM and nanoMOFs of 2.0 mg/mL. After different incubation times (30 min, 1 h, 2 h, 4 h, 6 h and 24 h), the suspensions were centrifuged and the supernatants were assessed by HPLC as previously described to determine the amount of released drug. Moreover, the trimesate release was also evaluated by HPLC. Briefly, trimesate was analyzed with a mobile phase consisting of 90% buffer (5.75 g/L of NH_4_H_2_PO_4_): 10% Acetonitrile containing 5 mM TBAP. The injection volume was 5 μl and the detection wavelength was set at 220 nm.

#### Human Plasma Protein Adhesion Tests

Human serum albumin (HSA) was used in this study. NanoMOFs modified or not (300 μg/mL) were incubated with HSA at 100 μg/mL in 10 mM phosphate buffer at 37°C. The samples were centrifuged at 10,000 *g* for 5 min to remove the nanoMOFs after 1, 2, 4, 6, 8 and 12 h incubation. The excessive amounts of HSA in the supernatant were quantified using a bicinchoninic acid (BCA) assay.

#### NanoMOF Internalization in Macrophage

NanoMOF internalization was quantified by Inductively Coupled Plasma Mass Spectrometry (ICP-MS). Macrophage cells (J774A.1) were seeded at a density of 2.0 × 10^5^ cells per well in 24-well plates. Cells were cultured at 37°C in 5% CO_2_ overnight for attachment. Cells were then incubated with 1 mL cell culture media containing nanoMOFs coated or not with lipids (nanoMOF concentration = 50 μg/mL). At the end of the 4 h incubation, the cells were washed with PBS for three times to eliminate the excess of MOFs. Cells were finally dried and digested using aqua regia (15 min under ultrasonic bath), Fe quantification was performed using an ICP-MS equipped with a triple quadrupole (Agilent 8800, Agilent Technologies, Japan). Fe and Co were added as internal standard on samples and calibration standards solution at a concentration of 10 μg/L. Isotopes were detected using “on-mass mode” (^54^Fe^+^, ^56^Fe^+^, ^59^Co^+^). Helium was introduced into the collision/reaction cell at a flow rate of 3 mL/min. Dwell time for each of the targeted isotopes was 1 s. Fe was quantified using external calibration prepared using certified 1000 mg/L Fe standard solution (Merck, Germany). Operation conditions were daily optimized using a tuning solution.

#### Cytotoxicity Assessment

MTT assays were carried out on SKOV3 ovarian cancer cell line to investigate the cytotoxicity of NPs. The cells were plated in 96 well plates at a concentration of 10,000 cells per well. The media was removed after 24 h incubation and replaced by fresh media containing the MOFs nanoparticles at different concentrations. The cytotoxicity was assessed by MTT assay at 24, 48 and 72 h following the incubation of the cells with the MOFs. In brief, 100 μL of complete media containing 0.5 mg/mL of MTT were added to cells and incubated for 2 h at 37°C in a 5% CO_2_ humidified atmosphere. Subsequently, the MTT media were removed and replaced by 100 μL of DMSO per well to dissolve the MTT-formazan crystals. The plates were shaken for 10 min at 350 rpm in a Heidolph Titramax 101 orbital shaker, and the absorbance at 595 nm was measured with the Tecan spark M10 plate reader. Each MTT experiment was reproduced three times.

## Results and Discussion

### MIL-100 (Fe) nanoMOF Surface Modification and Characterization of Functionalized NanoMOFs

**FIGURE 2:**
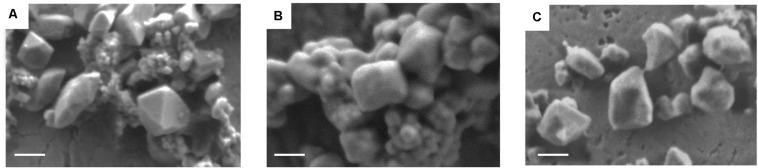
Representative scanning electron microscope (SEM) images of nanoMOFs before and after lipid modification. **(A)** nanoMOFs; **(B)** nanoMOFs after modification with DOPC; **(C)** nanoMOFs after modification with DOPC and DSPE-PEG 2000. Scale bar: 100 μm.

Iron trimesate nanoMOFs with mean diameters of 232 ± 14 nm and Brunauer–Emmett–Teller (BET) surface areas of 1519 ± 50 m^2^.g^–1^ were successfully synthesized by a “green” organic solvent-free hydrothermal method exempt of toxic additives such as hydrofluoric acid (Agostoni et al., 2013). They were crystalline and exhibited a facetted morphology (Figure 2A) in agreement with previously reported data (Cutrone et al., 2019a, b).

In an attempt to achieve “stealth” NPs, the as-synthesized nanoMOF were surface functionalized with PEG-lipid conjugates in a one-step procedure using a mixture of DSPE-PEG 2000 and DOPC. Lipids were associated within less than 2 min at room temperature by dispersing the nanoMOFs in an ethanolic aqueous solution containing both DSPE-PEG 2000 and DOPC, followed by a quick addition of water to favor lipid deposition onto the nanoMOF surface. Indeed, lipids were freely soluble in ethanol/water mixtures, but they readily precipitated upon progressive addition of water which drastically reduced their solubility, leading to precipitation onto the nanoMOF surfaces (Wuttke et al., 2015). DOPC-coated nanoMOFs were prepared as controls using the same method. The bare and coated nanoMOFs were characterized by a set of complementary methods.

Firstly, SEM images show that the lipid-coated nanoMOFs displayed similar shapes but with more rounded edges (Figure 2B) as compared to the uncoated ones (Figure 2A), possibly because surface modification. No significant differences were observed for the coated nanoMOFs with or without PEG-lipid conjugates (Figure 2C). Secondly, EDX experiments were performed to detect the presence of elements specific to the MOF cores (C, O, Fe) and to the shells (C, O, N) in the top layers of the NPs (around 10 nm depth). The presence of the DOPC coating was evidenced by the detection of an N peak characteristic of DOPC which was not found with bare nanoMOFs (Supplementary Figure S1). Interestingly, in the PEG shells obtained with the lipid mixtures, the relative O content was increased by a factor of 4 as compared to DOPC coatings (Supplementary Figure S1) possibly due to the presence of PEG chains in the nanoMOFs’ top layers, as PEG has the highest O content from all the nanoMOF components. These data offer a straightforward proof for both the presence of DOPC and PEG-lipid conjugates in the nanoMOF top layers.

**FIGURE 3:**
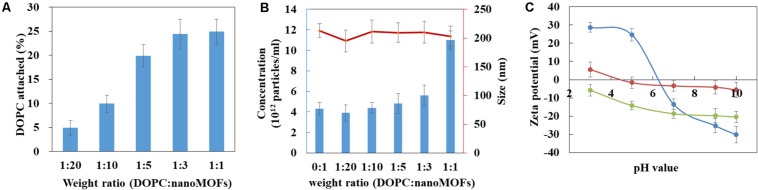
Characterization of lipid-coated nanoMOFs. **(A)** Quantification of the amount of DOPC in the nanoMOFs; **(B)** Mean hydrodynamic diameter (red) and concentration (blue) of DOPC-coated nanoMOFs determined by NTA. **(C)** Zeta potential of nanoMOFs as a function of pH before (blue) and after lipid coating with (red) or without (green) the addition of DSPE-PEG 2000.

The amount of DOPC in the nanoMOFs was quantified by using a colorimetric enzymatic method. For this, the DOPC:nanoMOF weight ratio in the preparation procedure was varied from 1:20 to 1:1. As shown in Figure 3A, the amount of lipids associated to the nanoMOFs increased with the amount of lipids used in the coating procedure. A plateau was reached at a DOPC: nanoMOF weight ratio of 1:3, corresponding to 25 ± 4 wt% lipids associated to the nanoMOFs. These quantities of coating material are among the highest reported so far (Horcajada et al., 2010; Agostoni et al., 2015; Bellido et al., 2015; Hidalgo et al., 2017; Giménez-marqués et al., 2018; Cutrone et al., 2019a, b). As comparison, phosphorylated cyclodextrin (CD-P) coatings on same iron trimesate nanoMOFs reached ∼ 17 wt% (Agostoni et al., 2015). The important lipid association could be possibly due to: i) the fast precipitation of lipids at the hydrophobic surface of nanoMOFs, and ii) the strong affinity of the phosphate groups in the lipids for the iron sites at the nanoMOFs’ surface.

The nanoMOFs, coated or not, were characterized by a set of complementary methods. First, X-ray powder diffraction (XRPD) showed that the crystalline structure of the nanoMOFs was preserved after surface modification (Supplementary Figure S2). Dynamic light scattering (DLS) proved that there were no significant differences between the mean hydrodynamic diameters of nanoMOFs before and after surface functionalization (232 ± 14 nm, 241 ± 17 nm and 238 ± 11 nm for uncoated nanoMOFs, lipid coated nanoMOFs at a DOPC: nanoMOF weight ratio of 1:3, with and without DSPE-PEG 2000, respectively). Moreover, the BET surface areas were not affected by surface modification with lipids (1519 ± 50 m^2^.g^–1^, 1486 ± 70 m^2^.g^–1^, and 1547 ± 80 m^2^.g^–1^ for uncoated nanoMOFs, lipid coated nanoMOFs with and without DSPE-PEG 2000, respectively), suggesting that the bulky lipids were located onto the nanoMOF’s external surfaces rather than into their porosity.

Before surface modification, the nanoMOF concentration was around (4 ± 0.8) × 10^12^ particles/mL, as determined by Nanoparticle Tracking Analysis (NTA). Interestingly, the nanoMOF particle concentration did not change upon modification with lipids (Figure 3B, blue histograms), suggesting that the lipids adhered at their surface and did not remain into the suspension medium. Indeed, at DOPC:nanoMOFs weight ratios from 0:1 up to 1:3, both particle concentrations and mean hydrodynamic diameters were unaffected [(5.6 ± 0.8) × 10^12^ particles/mL, and 210 ± 23 nm, respectively]. To support this hypothesis, DLS analysis of supernatants (Supplementary Table S1) after particle centrifugation revealed that they were devoid of any lipid vesicles (<1% particles free).

However, addition of excess lipids (weight DOPC:nanoMOF ratio of 1:1) resulted in a dramatic increase of total particle concentration [from (4 ± 0.8) × 10^12^ to (1.1 ± 0.4) × 10^13^ particles/mL], presumably because the nanoMOF surfaces were saturated with lipids. Of note, the mean hydrodynamic diameter of the nanoMOFs was unaffected, only the polydispersity index (PdI) increased from 0.15 to 0.25, possibly because of the presence of lipid vesicles in excess. Note that the association of DSPE-PEG 2000 didn’t significantly influence the mean hydrodynamic diameter, nor the nanoMOF’s concentration (less than 10% variations) suggesting that the PEGylated lipids also attached onto the nanoMOFs. In conclusion, lipids were associated up to 25 ± 4 wt% without inducing any changes in nanoMOF porosities, size distribution, and crystallinity.

Interestingly, the presence of the coatings affected the nanoMOFs electrophoretic mobility, as shown by Zeta potential (ZP) investigations in Figure 3C. Indeed, the ZP of the uncoated nanoMOFs was strongly dependent upon the pH of the suspension medium, shifting from positive values (+ 23 ± 3 mV) at pH lower than 5 to negative values (−15 ± 3 mV) at basic pH. This could be probably due to the presence of both uncoordinated iron sites and terminal carboxyl groups of the trimesate ligands at the external nanoMOFs surface (Cutrone et al., 2019b). The ZP values were dramatically altered after surface modification (Figure 3C). DOPC-coated nanoMOFs displayed negative ZP values (−6 to −20 mV) whatever the pH in the range of 3 to 10, in line with data reported for DOPC liposomes (Chibowski and Szcześ, 2016). These results support the presence of DOPC lipid layers onto the nanoMOFs which shield their charged surface moieties. Interestingly, when the nanoMOFs were surface-functionalized with PEG chains, their ZP values were shifted to neutral (–1.6 ± 3.4 mV). This is in good agreement with other studies on PEG-coated NPs (Gref et al., 1995; Thevenot et al., 2007; Troutier and Ladavière, 2007; Troutier-Thuilliez et al., 2009; Bugnicourt et al., 2019).

### Effect of the Coatings on the Colloidal Stability of NanoMOFs in Biological Media

**FIGURE 4:**
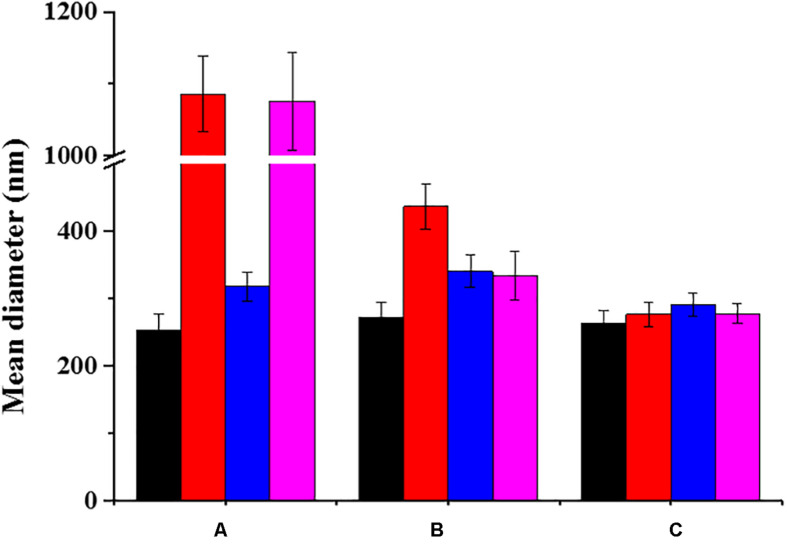
Colloidal stability of nanoMOFs in different media, before **(A)** and after surface functionalization with DOPC **(B)** or PEG-lipid conjugates **(C)**. Mean hydrodynamic diameters of nanoMOF suspensions at 100 μg/mL were determined by DLS after 6 h incubation at 37°C. (Black: water; red: PBS; blue: DMEM supplemented with 10v/v% FBS; pink: DMEM without FBS).

As the majority of uncoated NPs, nanoMOFs suffer from poor stability in biological media, which hampers their biomedical applications. Figure 4 clearly shows that uncoated nanoMOFs undergo a fast aggregation in both phosphate buffer saline (PBS, pH = 7.4, 10 mM) and cell culture medium DMEM (Dulbecco’s Modified Eagle Medium) without fetal bovine serum (FBS), with the mean hydrodynamic diameters rapidly increasing to more than 1 μm within only 6 h at 37°C (Figure 4). No significant variation was observed for the mean hydrodynamic diameter of uncoated nanoMOFs in water in the first 1 h, however, they tended to aggregate upon storage (Supplementary Figure S3). They were stable only in DMEM supplemented with 10% (v/v) FBS, possibly due to the formation of a protein corona at their surface preventing their aggregation (see section “Effect of surface functionalization of nanoMOFs on protein adsorption and macrophage uptake”).

In contrast, DOPC coated nanoMOFs were stable both in water and DMEM. No aggregation was observed even after 3 weeks storage. However, they still underwent aggregation in PBS (Figure 4). Remarkably, PEGylation allowed circumventing stability issues, whatever the suspension media (less than 10% diameter variation in PBS).

As all the coated and uncoated nanoMOFs were stable in DMEM supplemented with 10% (v/v) FBS, it was possible to explore further their cytotoxicity and interactions with cancer cell lines and macrophages. The PEGylated nanoMOFs exhibited excellent colloidal stability in all the tested biological media and thus appeared as optimal candidates for biological applications.

### Control of Degradation and Drug Release by Lipid Coating

There is a general agreement on the fact that once the nanomaterials release their drug cargo, they should degrade to avoid accumulation inside the body (Horcajada et al., 2012). However, Fe-based nanoMOFs are reported to degrade rapidly in the biological media, because of coordination of various ions (phosphates, sulfates, etc.) to their iron sites, sometimes leading to uncontrolled “burst” drug release (Agostoni et al., 2013; Li et al., 2017, 2019b). It was therefore interesting to investigate if the hydrophobic lipid coatings could interfere with the rapid penetration of the aqueous degrading media inside the pores, thus allowing gaining better control upon the release and degradation mechanisms.

**FIGURE 5:**
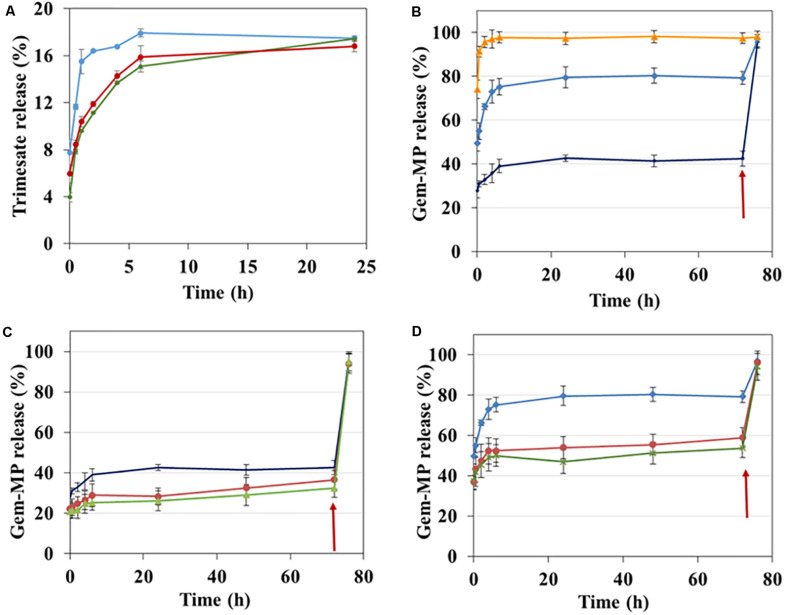
Effect of surface modification on Gem-MP and trimesate release analyzed by HPLC. **(A)** Trimesate release in 1 mM PBS from nanoMOFs before (blue) and after lipid coating with (red) or without (green) coating with PEG-lipid conjugates after incubation at 37°C. **(B)** Gem-MP release from uncoated nanoMOFs in PBS with different molarities (orange: 6 mM, blue: 3 mM; dark blue: 1 mM). Gem-MP release in 1 mM **(C)** or 3 mM **(D)** PBS from nanoMOFs before (blue/dark blue) and after lipid coating with (red) or without (green) coating with PEG-lipid conjugates. In all cases **(B–D)**, phosphate concentration was adjusted to 10 mM after 72 h incubation at 37°C (red arrows), followed by further incubation for 4 h at 37°C.

Degradation of nanoMOFs is generally monitored by the release of the constituting ligand trimesate (Agostoni et al., 2013; Rodriguez-Ruiz et al., 2015; Li et al., 2017). The degradation of the lipid-coated or bare nanoMOFs was studied by assaying ligand trimesate by HPLC in PBS (Figure 5A). In PBS 1 mM, uncoated nanoMOFs (blue curve, Figure 5A) underwent a fast degradation in the first 1 h at 37°C with around 15.5 ± 1.1% trimesate released, in agreement with previous reports (Li et al., 2017). It was discovered that in the same conditions, the lipid-coated nanoMOFs, with (red curve, Figure 5A) or without PEG-lipids conjugates (green curve, Figure 5A), exhibited much slower degradation profiles than the uncoated nanoMOFs, with only 10 ± 0.2% trimesate release in the first 1 h. This suggests a more progressive diffusion of the phosphate ions into the coated nanoMOFs, slowing down their degradation. However, the same plateau was reached after 24 h incubation, corresponding to a total complexation of the phosphates in the medium (Li et al., 2017). In conclusion, the shell efficiently delayed the degradation process.

Then, the effect of lipid coatings on drug release was studied. Selected drug of interest was Gem-MP, a hydrophilic drug with low cell permeability. NanoMOFs acted as efficient “nanosponges”, soaking Gem-MP from their aqueous solution with almost perfect efficiency (>98%). Maximal loadings reached 25 wt% reflecting the strong interaction between the drug and the iron trimesate matrices. Advantageously, the lipid coating process didn’t induce any significant drug release (less than 3% variations before and after coating).

Gem-MP release is governed by a competition of coordination between the phosphate moieties in Gem-MP and free phosphates in PBS for the iron(III) Lewis acids of nanoMOFs (Agostoni et al., 2013, 2015; Rodriguez-Ruiz et al., 2015). As expected, it was found that the higher the amount of phosphates, the higher the amount of drug released (Figure 5B). At low phosphate concentrations (PBS 1 mM or 3 mM), a plateau (around 40% or 80% Gem-MP release) was reached in 24 h, when all the phosphate molecules present in the release medium were complexed to the iron sites, as previously reported (Agostoni et al., 2013, 2015; Rodriguez-Ruiz et al., 2015). When additional phosphates were added in the release medium, all the drug still remaining in the nanoMOFs was immediately released (Figure 5B, arrow). Gem-MP release was well correlated with particle degradation, resulting in trimesate release (Supplementary Figure S4).

The presence of the lipid coating reduced the drug release from the nanoMOFs (Figure 5C). For instance, after 6 h incubation in PBS 1 mM, around 30% Gm-MP was released from the coated nanoMOFs, in comparison to 40% with the uncoated ones. This is possibly due to the restricted diffusion of phosphates into the nanoMOFs because of the lipid coating. Similarly, after 6 h incubation in PBS 3 mM, around 50% Gem-MP was released from the coated nanoMOFs, in comparison to 78% with the uncoated ones (Figure 5D). The Gem-MP release from coated nanoMOFs gradually increased in a sustained manner in the following days (Figures 5C,D). All the remained drugs could be released out after 4 h incubation in concentrated phosphate buffer (10 mM PBS).

**FIGURE 6:**
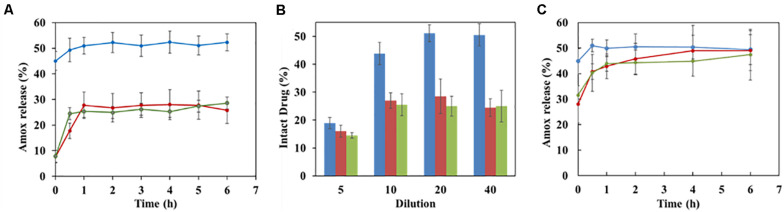
Effect of coating on Amox release in water **(A,B)** and in PBS **(C)**. **(A)** Release kinetics of Amox in water from nanoMOFs (1 mg/mL) before or after coating, with a dilution factor of 20; **(B)** Effect of dilution factor on Amox release after 4 h incubation at 37°C in water (Blue: uncoated nanoMOFs; red: DOPC coated nanoMOFs; Green: DOPC and PEG-lipid conjugate coated nanoMOFs).

Similar results were found with another drug, amoxicillin (Amox). The amount of Amox released in the first hour of incubation release was reduced by a factor of two in the case of lipid-coated nanoMOFs as compared to the naked ones (Figure 6A), confirmed by different dilution factors at 10, 20, and 40 (Figure 6B). However, in the presence of strongly complexing phosphates, the degradation was only delayed, but not avoided (Figure 6C).

### Cytotoxicity Assays of nanoMOFs on Ovarian Cancer Cells

**FIGURE 7:**
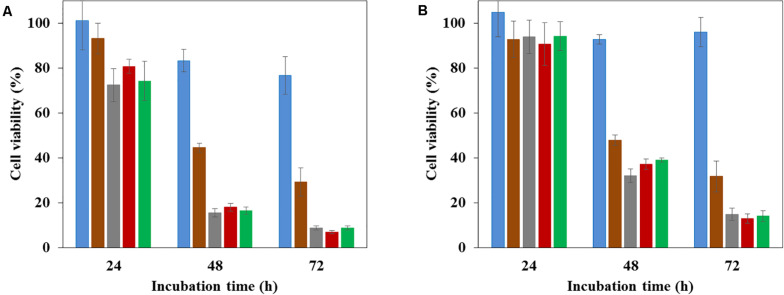
Cytotoxcity measured by MTT assays of nanoMOFs (blue), anticancer efficacy of Gem-MP (brown), Gem-MP loaded nanoMOFs before (gray) and after coating with DOPC (red) or PEG-lipid conjugates (green). The experiments were performed on SKOV3 ovarian cancer cells, at different incubation times, with different concentrations nanoMOFs. **(A)** 100 μg/mL; **(B)** 30 μg/mL. Gem-MP loading was 20 wt%.

All the studied nanoMOFs were non-toxic for the SKOV3 ovarian cancer cells up to 100 μg/mL (Figure 7A, blue histograms), with more than 98% cell viability in 24 h, which is in agreement with the previously reported lack of toxicity of these materials (Horcajada et al., 2010; Baati et al., 2013; Bellido et al., 2015; Giménez-marqués et al., 2018; Li et al., 2019b). In contrast, as expected, the anticancer drug Gem-MP (20 μg/mL, Figure 7A, brown histograms) exerted a cytotoxic effect with 45% cell viability after 48 h incubation, which further diminished to 29% in 72 h. Remarkably, Gem-MP loaded nanoMOFs showed a strong *in vitro* activity on SKOV3 ovarian cancer cells, higher than the free drug (Figure 7). At equivalent Gem-MP concentrations, whatever the drug loading (8 or 20 wt%) and the amount of nanoMOF in contact with the cells (10 to 100 μg/mL), the drug-loaded nanoMOFs outperformed the free drug in terms of toxicity on cancer cells (Figure 7 and Supplementary Figure S5).

This is in line with previous studies showing the efficient internalization of nanoMOFs bearing or not a lipid coating in pancreatic, breast, or bladder cancer cell lines (Rodriguez-Ruiz et al., 2015; Wuttke et al., 2015; Li et al., 2019a). It was recently shown that nanoMOFs acted as “Trojan horses” internalized by cancer cells, carrying their Gem-MP cargo to interfere with DNA (Li et al., 2019a). In this study it was shown that interestingly, the presence of a lipid coating (PEGylated or not) did not reduce the nanoMOF anticancer efficacy on SKOV3 ovarian cancer cells.

### Effect of Surface Functionalization of NanoMOFs on Protein Adsorption and Macrophage Uptake

It is well known that intravenously administered NPs are readily covered by plasma proteins, creating the so-called “protein corona”, which plays a crucial role on the NPs’ biodistribution and *in vivo* fate (Gref et al., 2000). To gain insight on the influence of lipid coating of nanoMOFs on protein adsorption, HSA (human serum albumin), the most abundant protein in human blood plasma, was selected for this study.

**FIGURE 8:**
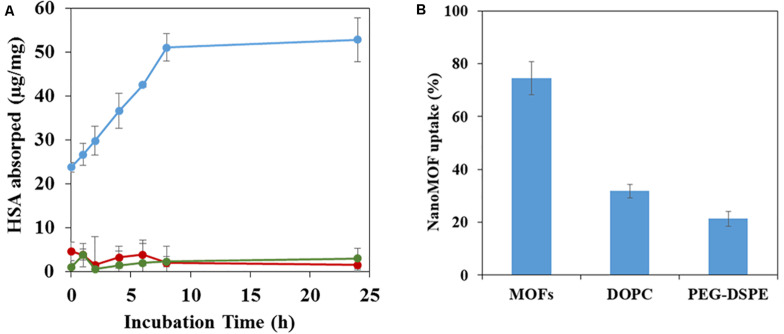
HSA adsorption assayed by BCA assay **(A)** and J774 murine macrophage uptake **(B)** of nanoMOFs before and after lipid surface functionalization. **(A)** HSA adsorption by nanoMOFs before (blue) and after lipid coating with (red) or without (green) the addition of DSPE-PEG 2000. **(B)** NanoMOFs (before and after lipid coating, with or without addition of DSPE-PEG 2000) internalization inside murine macrophage J774 cells. 50 μg/mL nanoMOFs were incubated with 3 × 10^5^ J774 cells for 4 h, and the amount of internalized nanoMOFs was determined by ICP-MS and expressed as a% of the initial nanoMOF amount in contact with the cells. Significant difference was observed for nanoMOFs before and after surface modification (*p* < 0.01).

NanoMOFs coated or not with DOPC lipids and PEG-lipid conjugates were incubated for 4 h at 37°C with HSA. After separation of the supernatants by centrifugation, the amount of non-adsorbed HSA was quantified using a BCA titration in order to determine the adsorbed HSA amounts onto nanoMOFs, lipid-modified or not. These amounts, expressed as μg/mg of nanoMOFs, are reported in Figure 8A. In the case of uncoated nanoMOFs (Figure 8A, blue curve), the amount of adsorbed HSA reached a plateau within 6 h, with around 50 μg HSA/mg nanoMOFs. Interestingly, lipid coating dramatically reduced HSA adsorption, to only ∼5 μg HSA/mg nanoMOFs (Figure 8A, green curve), regardless of the addition of DSPE-PEG 2000 (Figure 8A, red curve). To the best of our knowledge, these adsorbed HSA amounts are among the lowest reported with MIL-100 (Fe) nanoMOFs (Gref et al., 2000; Cutrone et al., 2019b), suggesting that lipid-based coating on nanoMOFs is efficient to avoid albumin adsorption.

The potential “stealth” effect of the lipid-coated nanoMOFs, PEGylated or not, was evaluated on the murine macrophage cell line J774. Quantitative data on the amounts of nanoMOFs internalized by cells were obtained by ICP-MS, after extensive washing to remove the non-associated particles. An incubation time of 4 h was chosen as it corresponds to the typical blood circulation time of PEG-coated NPs (Cutrone et al., 2019b). Interestingly, the DOPC coating of nanoMOFs reduced their macrophage uptake by a factor of 2.4, from 75 ± 6% to 31 ± 3% (Figure 8B). The nanoMOF functionalization with PEG chains was even more effective, reducing their interactions with macrophage to 21 ± 2%. Despite these great *in-vitro* results, it is widely known that numerous complex interactions can occur after NP administration in multicellular organisms. Therefore, *in vivo* studies need to be carried on to demonstrate the efficacy of the PEG coating to reduce reticuloendothelial system (RES) uptake.

Nevertheless, it has to be noted that, in similar experimental conditions, other coating materials showed higher interactions with macrophages, for instance, 41 ± 3% for CD-P coating, and 39 ∼ 24% for comb-like copolymers (Cutrone et al., 2019a, b). The advantage of lipid coating, demonstrated in this work, is a straightforward method, leading to efficient and stable coatings based on already FDA-approved materials. Lipid coatings on NPs are considered to be a promising strategy for the treatment of severe pathologies such as cancer (Luchini and Vitiello, 2019). In this study, the lipid coating not only afforded a control upon cell interaction but also provided a biocompatible protective barrier, modulating drug release and nanoMOF degradation. Of note, the nanoMOFs used in this study were shown to be biocompatible after intravenous administration in rats (Baati et al., 2013). However, the biocompatibility of the supermolecules assembled resulting from nanoMOFs coating with lipids has to be demonstrated *in vivo*.

## Conclusion

The surface of iron trimesate nanoMOFs was successfully modified with FDA approved DSPE-PEG 2000 in combination with DOPC by a fast solvent-exchange deposition method. We described herein the preparation and comprehensive characterization of the lipid modified NPs. We showed, for the first time, that the lipid surface modification of porous nanoMOFs reduced their tendency to degrade rapidly in PBS. Moreover, the coating of nanoMOFs with PEG-lipid conjugates successfully decreased their uptake by macrophages *in vitro* by a factor of 3.6. Finally, nanoMOFs acted as “Trojan horses” internalizing inside the cancer cells, and carrying their Gem-MP cargo to interfere with DNA.

## Data Availability Statement

The datasets generated for this study are available on request to the corresponding author.

## Author Contributions

RG conceived the study. RG, XL, and GS designed the experiments. XL, GS, and JQ preformed the experiments. CL contributed to the lipid investigations. XL and RG wrote the manuscript. MM, KB, and TT contributed to the biological evaluations. All authors approved the submitted version.

## Conflict of Interest

The authors declare that the research was conducted in the absence of any commercial or financial relationships that could be construed as a potential conflict of interest.
