# Supplementary material for: Drug-Loaded Lipid-Coated Hybrid Organic-Inorganic “Stealth” Nanoparticles for Cancer Therapy
Source: Front Bioeng Biotechnol. 2020 Sep 15;8:1027. doi: 10.3389/fbioe.2020.01027 (PMC7523570; doi:10.3389/fbioe.2020.01027)
Supplement: Supplementary file 1 [file Data_Sheet_1.docx]

Supplementary Material

Drug-loaded lipid-coated hybrid organic-inorganic “stealth” nanoparticles for cancer therapy

**Xue Li^1^, Giuseppina Salzano^1^, Jingwen Qiu^1^, Mathilde Menard^2^, Kristian Berg^2^, Theodossis Theodossiou^2^, Catherine Ladavière^3^, Ruxandra Gref ^1^***

^1^ Université Paris-Saclay, CNRS UMR 8214, Institut des Sciences Moléculaires d'Orsay, 91405, Orsay, France.

^2^ Department of Radiation Biology, Institute for Cancer Research, Norwegian Radium Hospital, Oslo University Hospital, 0372, Oslo, Norway.

^3^ University of Lyon, CNRS, UMR 5223, IMP, 15 bd André Latarjet, F-69622, Villeurbanne, France

*** Correspondence:**Ruxandra Gref
ruxandra.gref@universite-paris-saclay.fr


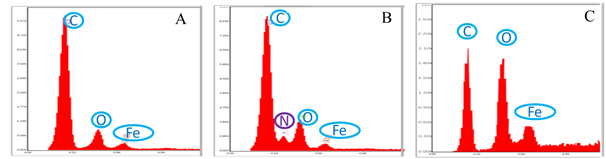


**Figure S1. EDX spectra of nanoMOFs before and after lipid surface modification.** A: Uncoated nanoMOFs; B: DOPC coated nanoMOFs; C: lipid coated nanoMOFs with the addition of DSPE-PEG 2000

Table S1 Mean hydrodynamic diameters of the NPs (characterized by DLS) in the supernatants obtained after centrifugation at 10,000 g for 10min of DOPC-coated nanoMOFs.

| No. | DOPC:nanoMOFs | Mean hydrodynamic diameter (nm) | Attenuator^a^ |
| --- | --- | --- | --- |
| 1 | 1:1 | 268 ± 26.4 | 9 |
| 2 | 1:3 | 153 ± 23.1 | 11 |
| 3 | 1:5 | 139 ± 25.3 | 11 |
| 4 | 1:10 | 146 ± 21.4 | 11 |
| 5 | 1:20 | 131 ± 24.3 | 11 |
| 6 | 0:1 | 143 ± 17.9 | 11 |

^a^ 11 is the maximal value of the attenuator


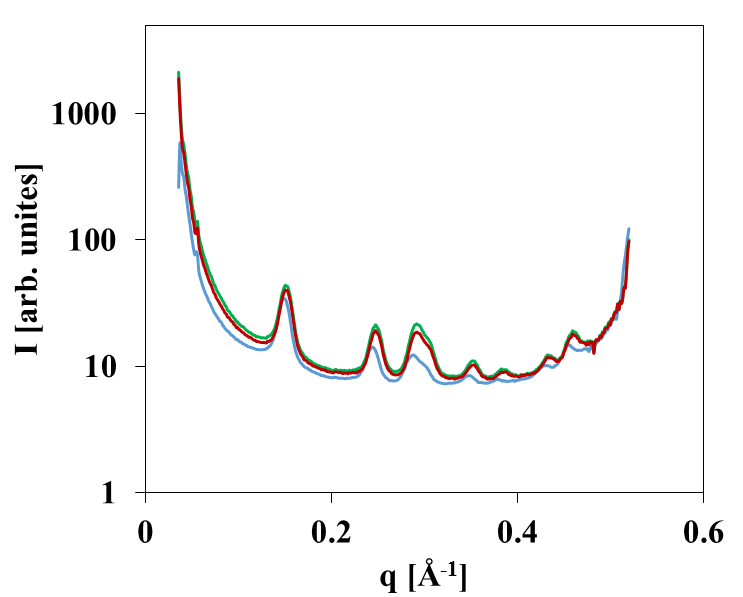


**Figure S2 Crystallinity of nanoMOFs before and after lipid surface modification.** XRPD patterns of uncoated nanoMOFs (blue), DOPC-coated nanoMOFs (green), and DOPC-coated nanoMOFs with the addition of DSPE-PEG 2000 (red).


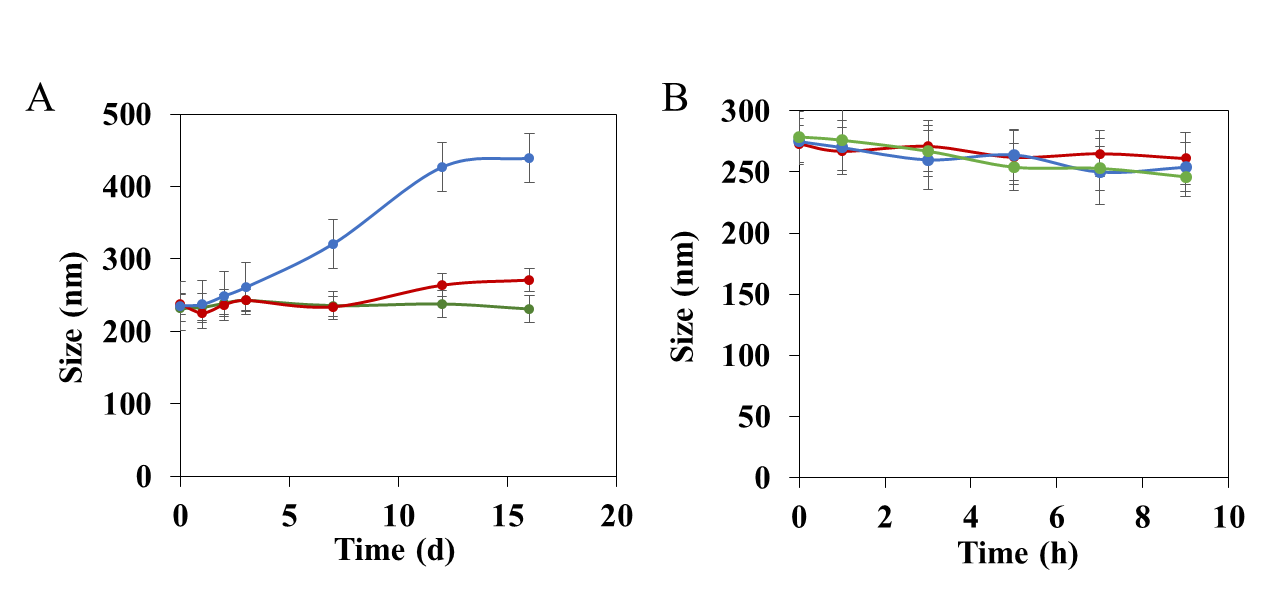


**Figure S3.** **Colloidal stability in water (A) and in cell culture medium (B) of nanoMOFs coated or not with lipids.** Blue: Uncoated nanoMOFs; Green: DOPC-coated nanoMOFs; Red: DOPC-coated nanoMOFs with the addition of DSPE-PEG 2000.


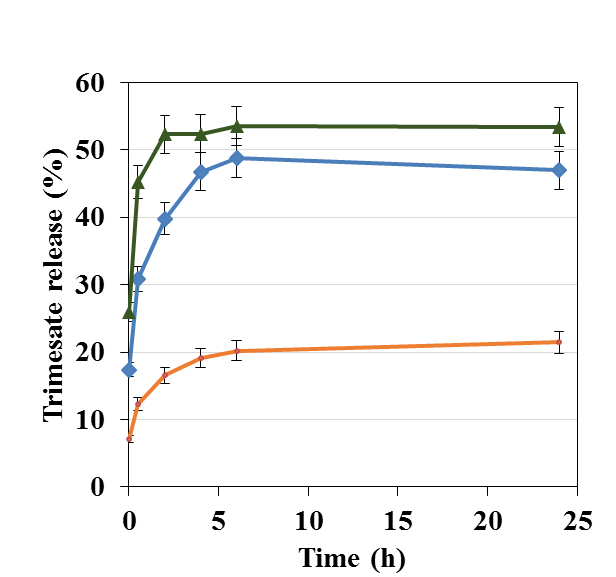


**Figure S4. Trimesate release of Gem-MP loaded nanoMOFs in PBS** (green: 6 mM; blue: 3 mM; orange: 1 mM)


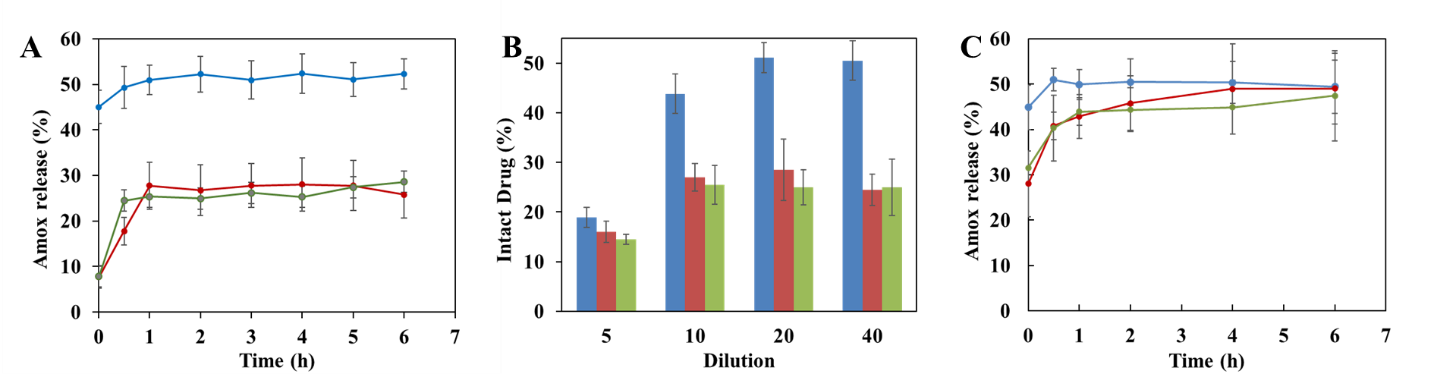


**Figure S5. Effect of coating on Amox release in water (A, B) and in PBS (C).** A: Release kinetics of Amox in water from nanoMOFs (1mg/mL) before or after coating, with a dilution factor of 20; B: Effect of dilution factor on Amox release after 4 h incubation at 37°C in water; (Blue: uncoated nanoMOFs; red: DOPC coated nanoMOFs; Green: DOPC and PEG-lipid conjugate coated nanoMOFs)


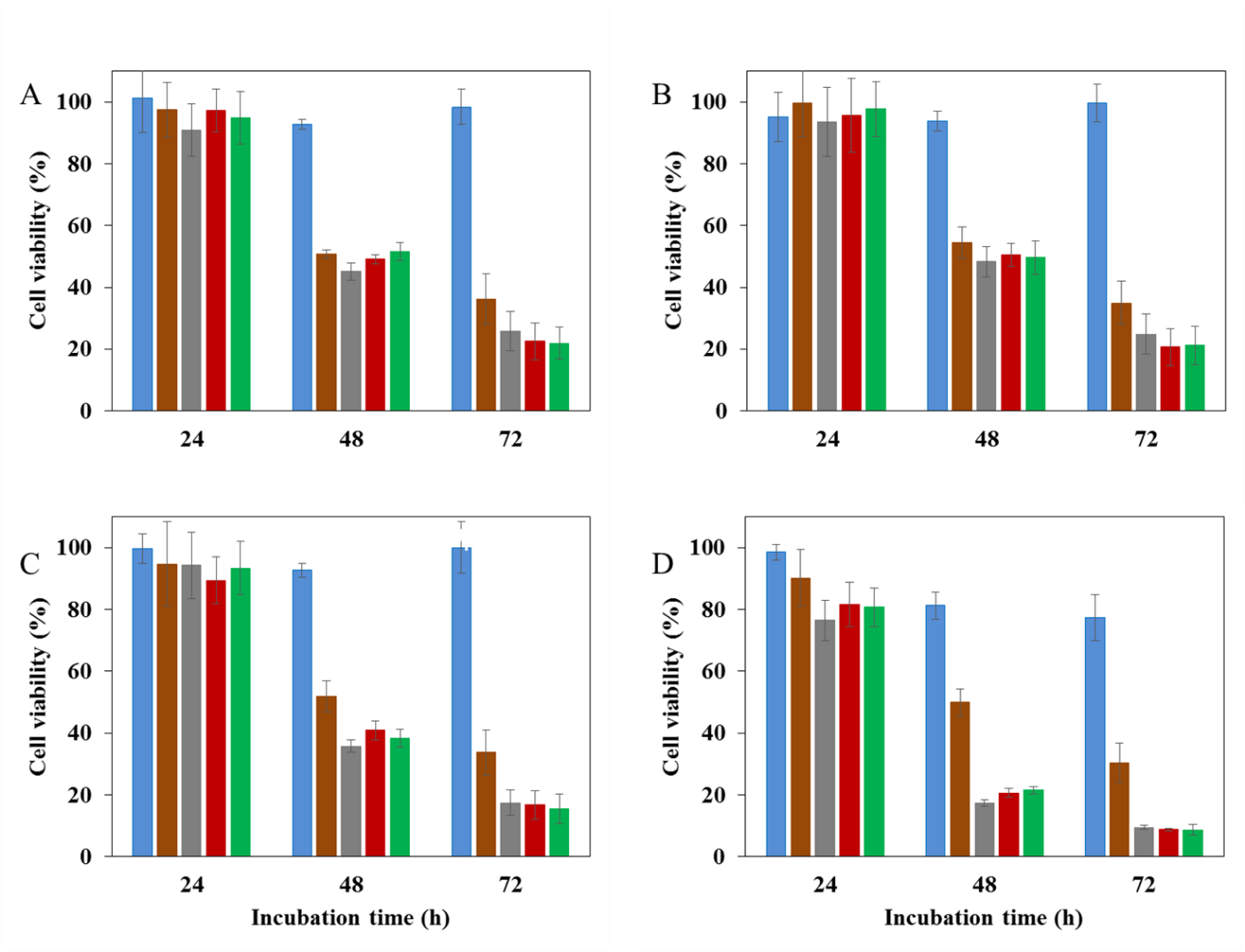


**Figure S6 Cytotoxicity of nanoMOFs (blue), anticancer efficacy of Gem-MP (orange), Gem-MP loaded nanoMOFs before (grey) and after coating with DOPC (red) or DOPC with PEG-lipid conjugates (green).** The experiments were performed on SKOV3 ovarian cancer cells. A: 10 µg/mL of 20% Gem-MP loaded nanoMOFs; B: 10 µg/mL of 8% Gem-MP loaded nanoMOFs; C: 30 µg/mL of 8% Gem-MP loaded nanoMOFs; D: 100 µg/mL of 8% Gem-MP loaded nanoMOFs.
